# Hemocyte siRNA uptake is increased by 5′ cholesterol-TEG addition in *Biomphalaria glabrata*, snail vector of schistosome

**DOI:** 10.7717/peerj.10895

**Published:** 2021-02-23

**Authors:** Anaïs Portet, Richard Galinier, Damien Lassalle, Alexandre Faille, Benjamin Gourbal, David Duval

**Affiliations:** 1 IHPE UMR 5244, CNRS, IFREMER, University of Montpellier, University of Perpignan, Perpignan, France; 2 Department of Medicine, Molecular Immunity Unit, University of Cambridge, Cambridge, United Kingdom; 3 Department of Haematology, University of Cambridge, Cambridge, United Kingdom; 4 Wellcome Trust-Medical Research Council Stem Cell Institute, University of Cambridge, Cambridge, United Kingdom; 5 Cambridge Institute for Medical Research, Cambridge, United Kingdom

**Keywords:** *Biomphalaria glabrata*, Cholesteryl TEG, RNAi interference, BgTEP1, Hemocyte

## Abstract

*Biomphalaria glabrata* is one of the snail intermediate hosts of *Schistosoma mansoni*, the causative agent of intestinal schistosomiasis disease. Numerous molecular studies using comparative approaches between susceptible and resistant snails to *S. mansoni* infection have helped identify numerous snail key candidates supporting such susceptible/resistant status. The functional approach using RNA interference (RNAi) remains crucial to validate the function of such candidates. CRISPR-Cas systems are still under development in many laboratories, and RNA interference remains the best tool to study *B. glabrata* snail genetics. Herein, we describe the use of modified small interfering RNA (siRNA) molecules to enhance cell delivery, especially into hemocytes, the snail immune cells. Modification of siRNA with 5′ Cholesteryl TriEthylene Glycol (Chol-TEG) promotes cellular uptake by hemocytes, nearly eightfold over that of unmodified siRNA. FACS analysis reveals that more than 50% of hemocytes have internalized Chol-TEG siRNA conjugated to Cy3 fluorophores, 2 hours only after *in vivo* injection into snails. Chol-TEG siRNA targeting BgTEP1 (ThioEster-containing Protein), a parasite binding protein, reduced BgTEP1 transcript expression by 70–80% compared to control. The level of BgTEP1 protein secreted in the hemolymph was also decreased. However, despite the BgTEP1 knock-down at both RNA and protein levels, snail compatibility with its sympatric parasite is not affected suggesting functional redundancy among the BgTEP genes family in snail-schistosoma interaction.

## Introduction

The fresh water snail *Biomphalaria glabrata* is one of the main vectors of the human flatworm parasite, *Schistosoma mansoni* in the Americas, responsible for Schistosomiasis, the second most widespread human parasitic disease after malaria (Batterman et al., 2009; Colley et al., 2014; Nelwan, 2019). As gastropods are essential for human schistosomiasis transmission, new strategies emphasizing on snail control to reduce schistosomiasis transmission in the field need to be developed. Among these vector control strategies, synthetic or natural chemical agent used as molluscicide (Augusto, Duval & Grunau, 2019; Coelho & Caldeira, 2016; Souza, 1995; WHO, 2019) and introduction of snail pathogens (Duval et al., 2015; Sandland, Rodgers & Minchella, 2007) or predators (Gashaw et al., 2008; Sokolow et al., 2015; Younes et al., 2017) into environment are investigated. Combating disease transmission requires a better understanding of the snail-parasite immunological interaction at the molecular level. In *B. glabrata/S. mansoni* interaction, a strain dependent compatibility polymorphism occurs (Theron et al., 2014). This compatibility gradient extends from totally incompatible interactions (i.e., resistant) to fully compatible interactions (i.e., susceptible). Such compatibility polymorphism arises from several genetic factors (Castillo et al., 2020; Coustau et al., 2015; Mitta et al., 2017; Pila et al., 2017; Portet et al., 2017), but can also be influenced by non-genetic factors modulated by environmental cues (Augusto, Duval & Grunau, 2019) such as temperature (Knight et al., 2015), pollution (Ibrahim, 2006) or UV exposure (Ruelas, Karentz & Sullivan, 2009). Deciphering molecular mechanisms by which snails and parasites interact appears essential to finding new ways for manipulating snail intermediate hosts (Tennessen et al., 2015) or for introducing in the field snails selected for their resistance alleles in order to decrease prevalence of human schistosomiasis (Coelho et al., 2008; Marques et al., 2014).

To validate the role of candidate genes that support the biochemical/molecular pathways involved in resistant and susceptible interactions, gene expression knockdown by RNA interference (RNAi) appeared as the widely used approach in invertebrate models (Lamacchia et al., 2013; Li et al., 2013; Li et al., 2020b; Robalino et al., 2007), and remains the best strategy available for functional validation in *Biomphalaria* snails, until CRISPR-Cas gene knockout development (Famakinde, 2018; Coelho et al., 2020). Indeed, there are few examples of CRISPR editing in gastropods to date (Abe & Kuroda, 2019; Perry & Henry, 2015), but their successful approaches pave the way to this technology development in closely related species. The first RNAi on *Biomphalaria sp.* was conducted to invalidate expression of Fibrinogen Related Protein 2 (FREP2), a lectin protein involved in snail immune response, with double-stranded RNA (dsRNA) and resulted in about 80% reduction of targeted transcript expression without revealing any particular phenotype (Jiang, Loker & Zhang, 2006). RNAi-mediated knockdown of FREP3 with a pool of siRNA induces a phenotype switch whereby some resistant snails became susceptible to *Echinostoma paraensei* (Hanington et al., 2010) or to *S. mansoni* (Hanington, Forys & Loker, 2012). The same strategy using combinations of siRNA duplexes was previously used to demonstrate the key role of FREPs in *Biomphalaria* innate immune memory, reducing the expression of the FREP2, 3 and 4 genes by 2- to 8-fold and rendered infected snails more susceptible to parasite (Pinaud et al., 2016). The RNAi strategy used also to demonstrate the key role of the cytokine BgMIF (Macrophage Migration Inhibitor Factor) or the growth factor BgGranulin in hemocyte proliferation and activation promoting parasite encapsulation respectively (Baeza Garcia et al., 2010; Pila et al., 2016a). The siRNA-mediated knockdown of BgTLR (Toll-Like Receptor), a transmembrane protein expressed in some hemocytes showed defective phagocytosis and partially suppressed the resistance phenotype of BS90 snails (Pila et al., 2016b). All the studies described above show an injection into the pericardial cavity of *B. glabrata* snails of naked interfering RNA molecules (Baron et al., 2013) or incubated with transfection reagent (Allan et al., 2017; Pila et al., 2016a). A non-invasive siRNA delivery has been successfully developed by soaking juvenile snails in PEI (polyethylenimine) a cationic polymer (Knight et al., 2011). Despite all these experiments, the nature and number of transfected cells in snails remain unknown.

In the present study, we used a siRNA targeting BgTEP1, a thioester-containing protein mainly expressed in hemocytes (Portet et al., 2018), the snail immune cells. It has been suggested that BgTEP1 could play an opsonin role, binding *S. mansoni* sporocyst surface (Portet et al., 2018). It was also demonstrated that BgTEP1 is able to bind an immune complex formed by SmPoMuc (*Schistosoma mansoni* Polymorphic Mucins) and FREPs (Li et al., 2020a; Mone et al., 2010). Herein we focused on siRNA delivery into hemocytes for efficient gene silencing in vivo. To achieve this, we designed a chemical modification on the 5′ end of siRNA by conjugation with a Cholesteryl-TEG (Triethylene Glycol). This lipophilic addition has been shown to be beneficial to increase siRNA uptake by HeLa cells (Shmushkovich et al., 2018). First, modified and unmodified Cy3- labeled siRNA were compared in hemocytes ex vivo collected from snails to monitor siRNA delivery. Then, flow cytometry analysis was conducted to follow delivery efficiency in hemocytes in-vivo after Chol-TEG siRNA injection into living snails. Finally, silencing of BgTEP1 transcript target, using cholesteryl-TEG siRNA was done. The efficiency of BgTEP1 silencing was assessed at both RNA and protein levels by RT-qPCR and western blot. Then BgTEP1-siRNA treated snails were exposed to miracidia of *S. mansoni* to monitor any changes in infection phenotype.

## Materials and Methods

### Biological materials

*B. glabrata* originated from Guadeloupe (BgGUA2) and its sympatric strain of *S. mansoni* (SmGH2) were used for experimental approaches. BgGUA2 was provided from M. Blouin (Oregon State University) in 2013. These snails were collected in 2005 from Dans Fond on the island of Guadeloupe (DFO; N:16°18.500′, W:061°30.720′). For infection experiments, each snail was individually exposed for 12 h to 10 miracidia in 5 mL of pond water. Breeding conditions and maintenance of parasite cycle have been conducted as previously described (Portet et al., 2019).

The laboratory and experimenters possessed an official certificate from the French Ministry of National Education, Research, and Technology, CNRS and DRAAF Languedoc Roussillon for experiments on animals, animal housing, and animal breeding (#A66040; decree # 87–848, October 19, 1987; and authorization # 007083).

### siRNA uptake and interference

Small interfering RNA duplexes against BgTEP1 and Green Fluorescent Protein (GFP used as control) were synthetized and modified by Eurogentec Company and purified by standard *SePOP* desalting. In this study, two siRNA targeting BgTEP1 transcripts mRNA (GenBank accession No. FJ480411) which are 5′ GAC-AGA-UUC- UCA-UCA-AAC-A and 5′ GAG-UAU-GAU-UUA-CCA-AGA-U and one against the cnidarian GFP (GenBank accession No. EU430082): 5′ CAA-GCU-GAC-CCU-GAA-GUU-C were synthetized. The GFP siRNA was designed with and without a Cholesteryl-TEG added in 5′ to assess if this modification can increase siRNA cellular uptake. A Cy3 dye was linked to the 3′ termini of each RNA molecule for siRNA duplex in order to follow cellular uptake by flow cytometry approach. To investigate the silencing effect on the BgTEP1 mRNA level and on snail susceptibility phenotype to *S. mansoni*, the different siRNAs (BgTEP and GFP) were designed only with a Cholesteryl-TEG added in 5′ end. Two siRNA targeting BgTEP1 transcripts suppress mRNA were thus injected in equimolar quantity in the same snails. Each duplex siRNA was annealed and shipped dried by the manufacture (Eurogentec). siRNA was suspended in sterile Chernin’s balanced salt solution (CBSS). Concentrations of siRNA were adjusted so injected volumes do not exceed 5 µl. Each siRNA was injected into the cardiac sinus of *B. glabrata* snails (7–8 mm in diameter), using a 50 µl Hamilton syringe with a 26s needle (Hamilton) (Baeza Garcia et al., 2010).

### siRNA delivery into hemocytes ex vivo

500 µL of hemolymph were recovered by head-foot retraction from pool of 3 adult snails, transferred to a 1.5 ml microcentrifuge low DNA binding tube and then incubated with 2 µg of Cy3-labeled GFP siRNA with and without Chol-TEG at 24 °C after mixed by gentle inversion. siRNA delivery was monitored from 30 min to 1, 2, 4, 6 and 8 h post-incubation by flow cytometry analysis (see FACS analysis section). For each experimental point, 4 biological replicates were performed. For comparing the percentage of fluorescent cells following the different treatments a Fisher Exact test was used, results were considered significant for *P* value ≤ 0.05. For comparison of siRNA incorporation over the kinetic after injection, a Kruskall–Wallis with Dunn post-hoc tests were used to test significant differences (*P* value ≤ 0.05) between experimental conditions.

### siRNA delivery on hemocytes in vivo

2 µg of Cy3-labeled Chol-TEG siRNA targeted GFP (500 ng/µl) were injected in the snail pericardial cavity. Cellular uptake of siRNA was evaluated at 2, 4, 6, 12 and 24 h after injection (*n* = 4 snails for each condition). The hemolymph was recovered as described above. A negative control consisting in analysing the hemocyte fluorescence from uninjected snails was also performed. Then, a similar approach was conducted by varying the amount of siRNA injected to snails (2, 5 and 10 µg). Hemolymph was recovered 2 h after injection from 4 individual snails used as biological replicates. Significant differences in kinetic of siRNA cellular uptake following injection were tested using Kruskall-Wallis followed by Dunn post-hoc test (*P* value ≤ 0.05). Significant differences between doses of siRNA were tested using a Mann–Whitney *U* test. Results were considered significant when *P* value ≤ 0.05.

### Flow cytometry analysis

After both in vivo and in vitro siRNA delivery, 100 µL of hemolymph were fixed with 100 µL of 4% paraformaldehyde solution in PBS for 5 min. Then, hemocytes were collected after centrifugation at 1,000 rpm, 10 min and re-suspended in PBS snail as previously described (Pinaud et al., 2016). Hemocyte preparations were stored at 4 °C and analysed one day after storage. The Flow Cytometry was performed using a FACS Canto BD Biosciences (RIO Imaging Platform, Montpellier, France). For each sample, around 10,000 events were counted. The results were analysed and visualised using the FlowJo V 10.0.8 software. To demonstrate internalization of cholesteryl-TEG siRNA into hemocytes, 500 µL of hemolymph from pool of 3 adult snails were plated for 1 h on polystyrene chamber slides. Then, hemocytes were incubated 2 h with 2 µg of Cy3-labeled siRNA. Hemocytes were washed twice in PBS and fixed in 4% paraformaldehyde for 5 min. After rinsing with PBS, actin filaments were stained with phalloidin  conjugated with Alexa 488 (Thermo Fisher) for 15 min and the cell nucleus with DAPI (Biotum) for 30 seconds as previously described (Portet et al., 2018). After rinsing, slides were mounted in Dako fluorescent mounting medium (Dako) and examined using a fluorescence confocal laser-scanning microscope (Zeiss LSM 700, Bio-Environment platform). For presentation, images were imported into ImageJ software.

### RNA extraction and Quantitative RT-PCR analysis

Using HPLC syringe, 10 µg of Chol-TEG siRNAs targeting BgTEP1 (quantity ratio 1:1) or GFP were injected in snail pericardial cavity (2µg/µl). Hemolymph was recovered at 1, 2, 3 and 4 days after injection from 4 independent pools of 3 snails. Hemocytes were recovered by centrifugation and hemocyte total RNA was then extracted using the Total RNA Purification Micro Kit (Norgen Biotek) according to the manufacturer’s instructions. Reverse transcription was performed from 100 ng of RNA with oligo dT using Maxima H Minus First Strand cDNA Synthesis Kit with dsDNase (Thermo Scientific, Whaltham, Massachussetts, USA) according to manufacturer’s instructions. Quantitative PCR (Q-PCR) was performed using NO ROX SYBR® Master Mix blue dTTP (Takyon) and run on LightCycler 480 thermocycler (Roche) according to the manufacturer’s instructions. Sequence of primers used for BgTEP1 amplification (forward: 5′ CGT-ACT-TAC-CCT-CGC-TC, reverse: 5′ ACC-ATT-AGA-TCC-ACT-GGA-AGA-TA) and ribosomal protein S19 (accession number: XM_013211381) (forward: 5′ TTC-TGT-TGC-TCG-CCA-C, reverse: 5′ CCT-GTA-TTT-GCA-TCC-TGT-T) were designed using LightCycler Probe Design software version 1.0 (Roche Diagnostics). 2 µl of cDNA diluted to 1/20 in ultrapure water was used in a reaction mixture containing 0.1 µM of each primer. The cycling program was as follows: denaturation step at 95 °C for 2 min, 40 cycles of amplification (denaturation at 95 °C for 10 s, annealing and elongation at 60 °C for 20 s), with a final step at 60 °C for 5 min. QPCR was ended by a melting curve step from 65 to 97 °C with a heating rate of 0.11 °C/s and continuous fluorescence measurement. The cycle threshold (*C*_*t*_) was determined using the second derivative method of the LightCycler 480 Software release 1.5 (Roche). Sequence of PCR amplicon was checked by Sanger sequencing. The relative expression of BgTEP1 was calculated with the ΔΔCt method as the efficiency of both couple of primers presented the same PCR amplification efficiency. Briefly, BgTEP1 expression was normalised to the endogenous control, the ribosomal protein S19, for each condition (ΔCt, Ct_BgTEP1_-Ct_S19_). Then, the relative expression of BgTEP1 was determined by the ΔΔCt method based on this expression of the nonsilencing control group (ΔΔCt, ΔCt_(siRNA injected)_ − ΔCt_(untreated snails)_). For graphical representation, relative expression data presented are determined as the ratio between relative expression of BgTEP1 with respect to S19 level under BgTEP and GFP interference condition (Pfaffl, 2001; Knight et al., 2011). Kruskall-Wallis with Dunn post-hoc tests were used to test significant differences (*P* value ≤ 0.05) between experimental conditions.

### BgTEP1 protein expression level analysis

Using the procedure described previously, 10 µg of Chol-TEG BgTEP1 siRNA were injected in 4 replicates of 3 snails for each condition. Cell free hemolymph was collected 1, 2, 3 and 4 days post injection and protein content was determined using 2-D Quant Kit (GE Healthcare). Five microliters (15 µg total protein/µL of hemolymph) of hemolymph were denatured 5 min at 99 °C in 1X Laemmli buffer plus *β*-mercaptoethanol, before proteins separation on 7.5% SDS-polyacrylamide gel and transferred onto a 0.2 µm Hybond ECL membrane (GE Healthcare) using a Transblot transfer system (Bio-Rad). After saturation over a period of 3 h at room temperature in TBSTM [1 × TBS (500 mM Tris-HCl, 1.5 M NaCl, pH 7.5), 0.05% Tween-20, 5% non-fat milk], the protein blots were incubated overnight at 4 °C in TBSTM, with a 1:5,000 dilution of the rat polyclonal anti-BgTEP1-N-ter antibody (Agro-Bio), raised against the recombinant BgTEP1 N-terminal part (amino acid 204 to 521 from BgTEP1 protein sequence (GenBank ID QEQ12617)). The blots were washed three times with TBST, and further incubated with 1:5,000 dilution of the commercial horseradish peroxidase-conjugated rabbit anti-rat IgG (H + L) antibody (CliniSciences #6180-05). Blots were finally washed three times with TBST, and one time with TBS, and then revealed in the presence of an enhanced chemiluminescent substrate (SuperSignal West Pico Plus Chemiluminescent Substrate, Thermo Scientific). Image acquisition was performed using ChemiDoc MP Imaging System (Bio-Rad) for 3 s. Blot picture and relative quantitative analysis of bands were performed using Image Lab Software version 4.0.1.

Biomphalysin was used as internal control for protein loading and to exclude a protein degradation. A western blot on the same samples was performed according to the same procedure used for BgTEP1 protein detection. To reveal Biomphalysin in the cell-free hemolymph, a polyclonal antibody raised against this toxin (Agro-Bio) and an HRP-conjugated goat anti-rabbit IgG secondary antibody (ImmunoPure Antibody, Pierce) were diluted at 1:1,000 and 1:7000 respectively in TBST.

### Effect of BgTEP1 mRNA silencing on *S. mansoni* infection phenotype

BgGUA2 snails were injected with 10 µg of Chol-TEG siRNA duplexes specific for BgTEP1 (43 snails) and GFP (34 snails) and individually exposed for 12 h to 10 *S. mansoni* GH2 miracidia in 5 ml of pond water at 3 days after siRNA injection. Then, 16 days after parasite exposure, the snails were fixed in Raillet-Henry solution. The prevalence (P: % of snail infected) and the intensity (I: number of parasites per infected snail) were determined by counting the primary sporocysts present in each snail tissue as described previously (Portela et al., 2013). Briefly, the snails were relaxed in water containing an excess of crystalline menthol for 6 h. The snail shell was removed and the body was fixed in modified Raillet-Henry’s solution (930 ml distilled water, 6g sodium chloride, 50ml formol 40%, 20 ml 95 acetic acid). The presence of Sp1 in each snail was numbered by visual inspection and meticulous dissection of the snail tissues. Following Raillet-Henry’s fixation the Sp1 were readily observable as opaque white bodies within a yellow snail tissue background. Significant changes in prevalence and intensity were tested by Fisher exact test and Mann–Whitney *U* test.

## Results

### Ex vivo siRNA delivery into hemocyte is enhanced by lipid-conjugation

**Figure 1:**
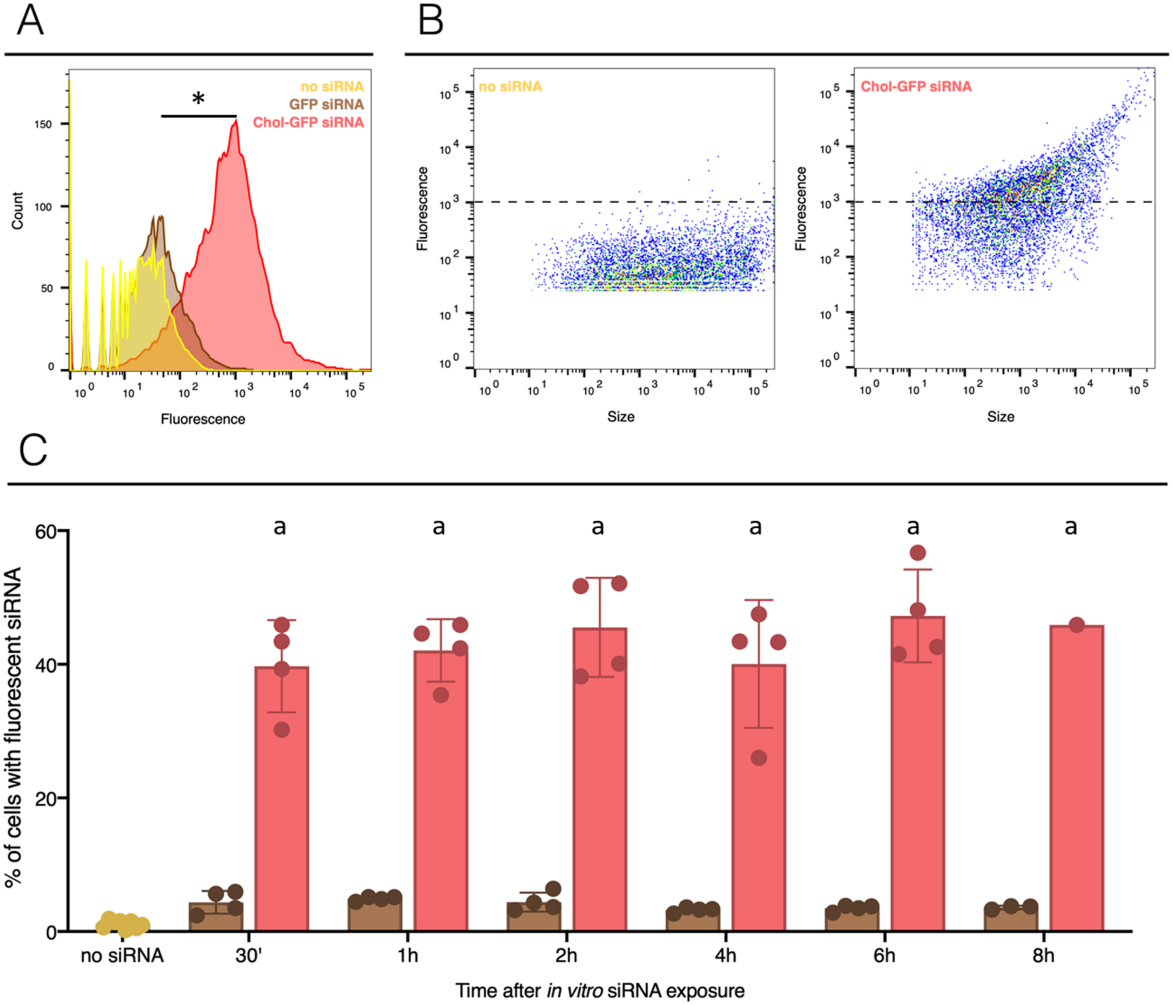
siRNA incorporation with or without lipid-conjugation in hemocytes *ex vivo*. The no siRNA condition is yellow, GFP siRNA is brown and GFP siRNA with Cholesterol-TEG conjugation (Chol-GFP siRNA) is red. (A) Fluorescence profile in the three conditions, count corresponds to the cells numbers and the fluorescence scale corresponds to the fluorescence signal uptake value. Negative control or unexposed cells to siRNA (named: no siRNA) was used to determine the levels of auto-fluorescence (limit 10^3^). (B) Profile of hemocytes according to their fluorescent labeled siRNA uptake and their size. The dotted lines indicate to the limit of auto-fluorescence. (C) Flow cytometry analysis of siRNA uptake by hemocytes after in vitro injection of 2 µg of GFP siRNA (with or without Chol-TEG). The naive point corresponds to cell auto-fluorescence. The “*” indicate significant difference in the % of cells with fluorescent siRNA comparing control (no siRNA), GFP siRNA and Chol-GFP siRNA (Fisher exact test, *p* < 0.05) (Fig. 1A). Means with different letters are significantly different (Kruskall-Wallis test with Dunn post-hoc further adjusted by the Benjamini-Hochberg FDR were used, *p* < 0.05) (Fig. 1C).

To assess the delivery of siRNA conjugated to cholesterol in snail hemocytes, naked and modified (Chol-TEG) GFP-siRNA were coupled with the fluorochrome Cy3. Two micrograms of labelled GFP-siRNA were added to 500 µL of freshly collected hemolymph from a pool of adult snails. Monitoring of cell uptake by FACS revealed that siRNA were quickly internalized by hemocytes in both conditions and showed a high number of fluorescent cells after 30 min of exposition (Fig. 1 and Fig. S1). More siRNA were uptaken by cells when cholesterol was conjugated (Figs. 1A and 1B), 47% of cells were counted with a cytosolic fluorescence compared to 5% of cells when using unmodified siRNA (Fisher exact test, *p* = 0.00001, *n* = 4 per group). No significant differences were observed when comparing unmodified siRNA to untreated snails hemocyte auto-fluorescence (Fisher exact test, *p* = 0.3687, *n* = 4 for unmodified siRNA and *n* = 8 for control). A closing parenthesis has been deleted here: [(Fisher exact test,...for control)]. Please check, and correct if necessary (Fig. 1C). Cholesterol conjugation enhances significantly the cellular uptake of siRNA. Also, internalization of siRNA did not increase over time in vitro condition; the number of fluorescent hemocytes remains similar after 30 min or 8 h exposure time for both conditions (GFP-cy3 Kruskal-Wallis test *p* = 0.145552, *df* = 5, *n* = 24, *H* = 8.200 ; Chol-TEG-GFP-cy3 Kruskal-Wallis test *p* = 0.643170, *df* = 5, *n* = 24, *H* = 3.369826, *n* = 4 per group) (Fig. 1C). Therefore, Chol-TEG siRNA was used for subsequent experiments.

### In vivo siRNA delivery into hemocyte

**Figure 2:**
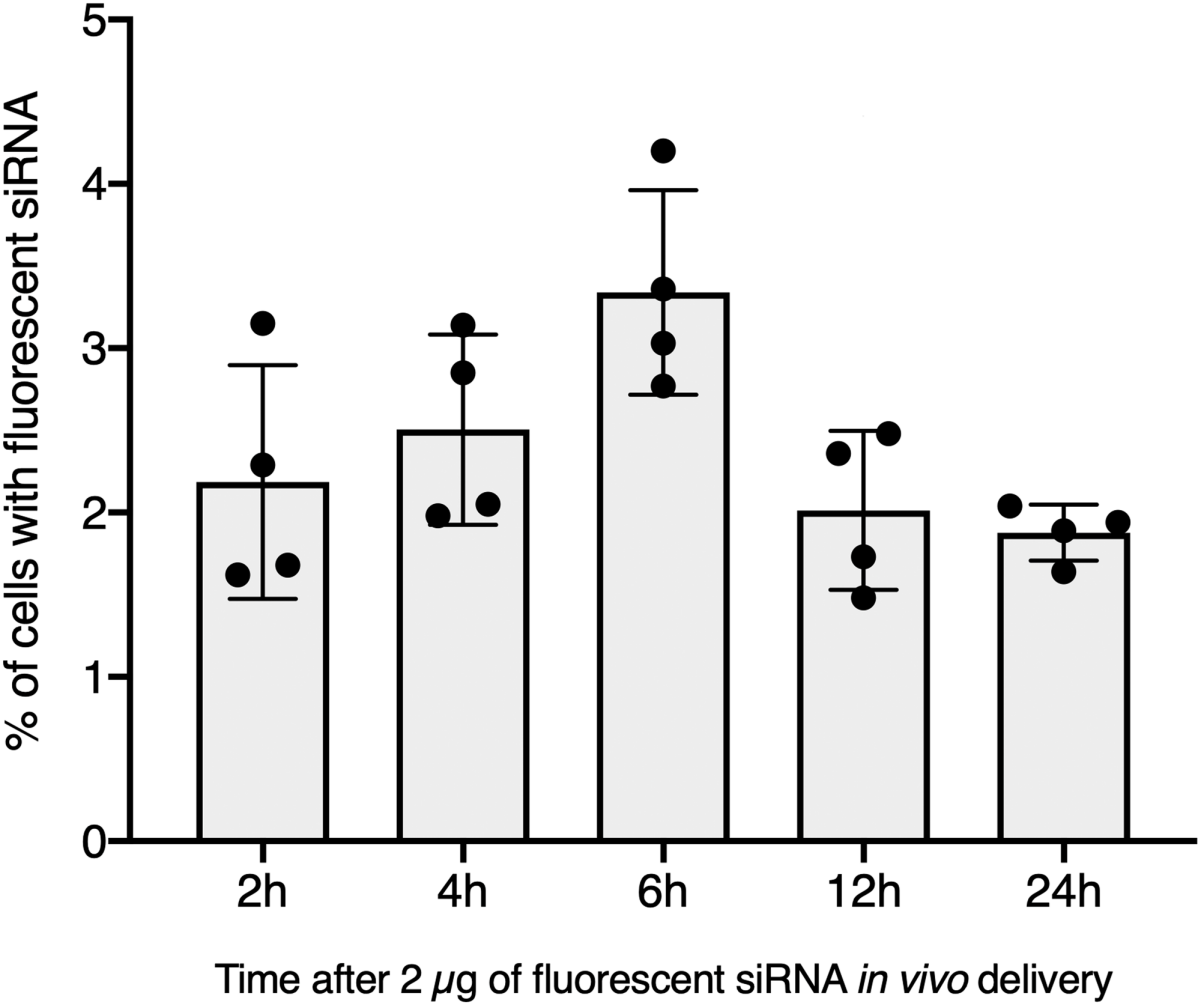
In vivo hemocyte incorporation of Cholesteryl-TEG siRNA. Flow cytometry analysis of siRNA uptake by hemocyte following in vivo injection of 2 µg of Chol-GFP siRNA along a time serie analysis. No significant differences between time points were observed (Kruskall-Wallis test followed by a Dunn post-hoc test further adjusted by the Benjamini-Hochberg FDR method were used).

**Figure 3:**
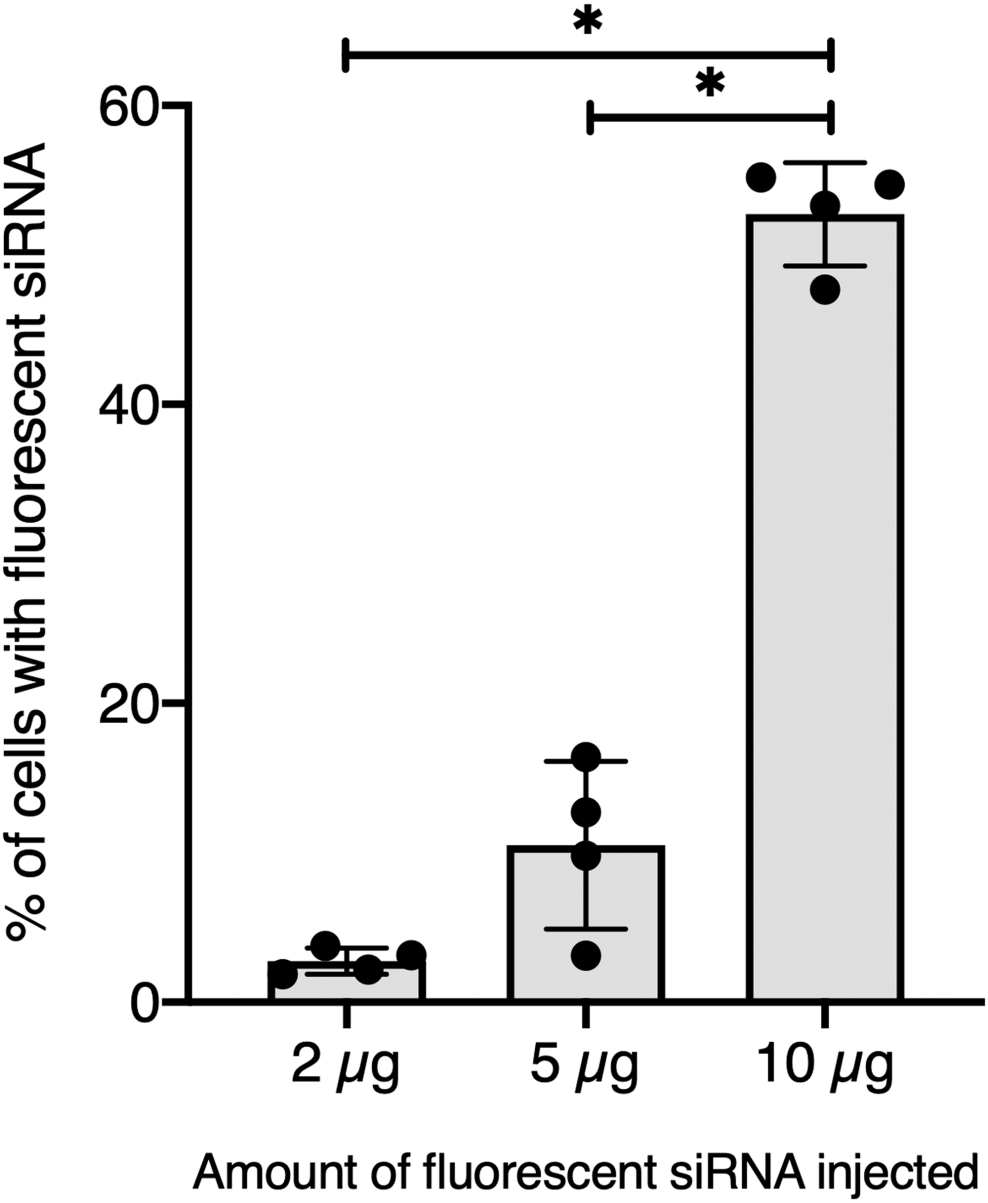
Quantitative optimization of siRNA injected into snails for an efficient delivery on hemocyte in vivo. Flow cytometry analysis of siRNA uptake by hemocyte 2 hours post in vivo injection of different doses of Chol-GFP siRNA. The “*” corresponds to significant differences between two different time points (Mann-Whitney U test was used).

To further validate our GFP-siRNA delivery condition in vivo, 2 µg of Chol-TEG-siRNA against GFP mRNA were injected in snail pericardial cavity. Flow cytometry analysis was performed on hemolymph recovered between 2 h and 24 h post-injection to follow hemocyte uptake efficiency in vivo. Overall, the percentage of internalized Chol-TEG-siRNA is much lower than that obtained in vitro. Indeed, only 1.9 to 3.3% of hemocytes have incorporated Chol-TEG-siRNA regardless of exposure time (no significant differences over time: Kruskal-Wallis test *p* = 0.062544, *df* = 4, *n* = 20, *H* = 8.9428) (Fig. 2) compared to around 50% for in-vitro condition after 30 min (Fig. 1C). Therefore, to optimize cellular uptake, increasing amounts of siRNA have been injected into snails and hemocytes were recovered 2 h post-injection. The proportion of fluorescent cells after 2 h post-injection increased with higher Chol-TEG siRNA concentrations (Fig. 3). Hemocytes have internalized significantly more Chol-TEG-siRNA from 11% or 55% after the injection of 5 µg or 10 µg respectively (Mann–Whitney *U-* test, *U* = 0 ; *z*-score = −2.50672 ; *P* = 0.00604, *n* = 4 per condition) (Fig. 3). Consequently, we decided to use a dose of 10 µg of siRNA coupled with Cholesteryl-TEG for further analysis.

### Knockdown of BgTEP expression in guadeloupean snails

**Figure 4:**
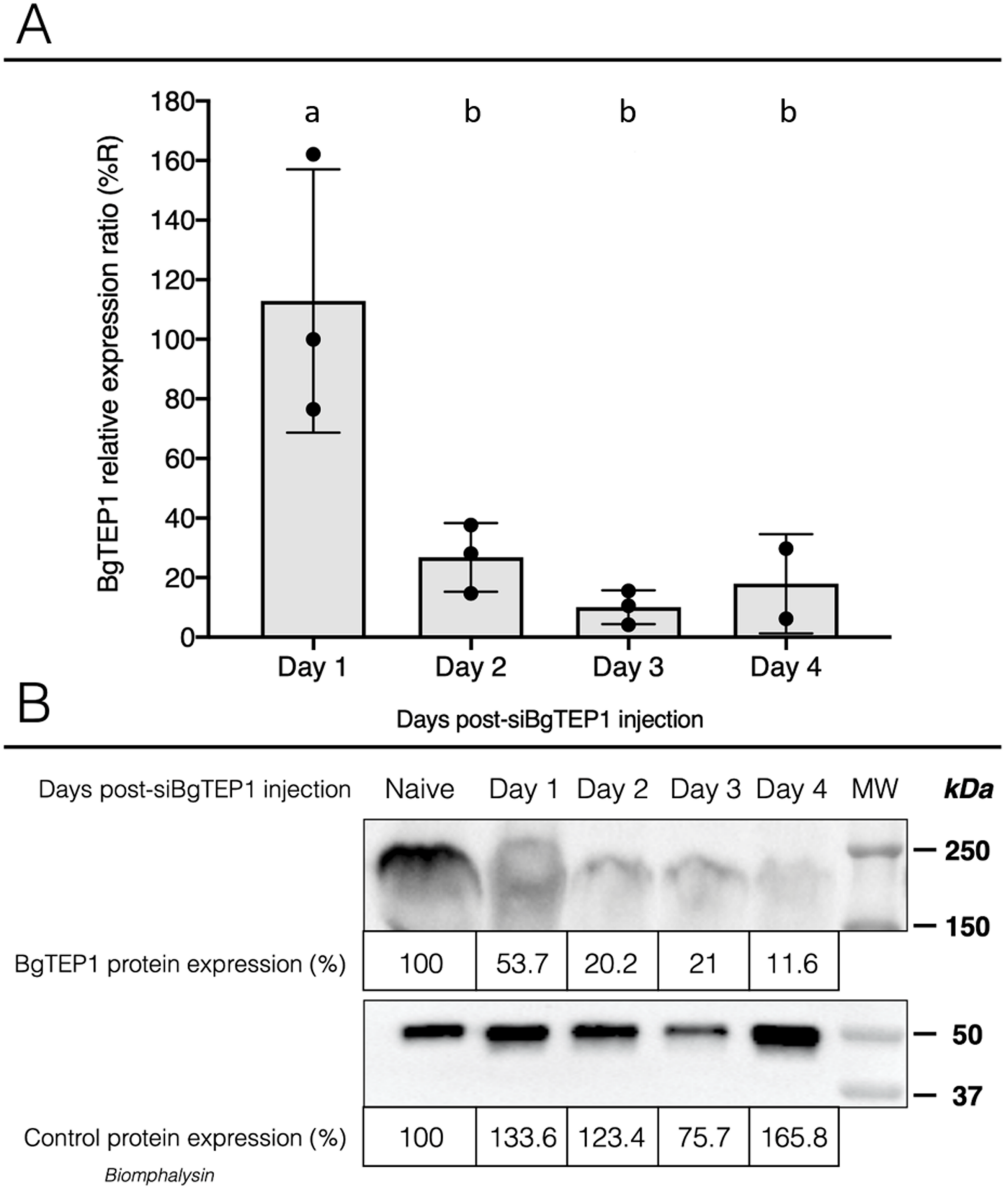
Knockdown of BgTEP1 expression in guadeloupean snails. (A) Relative expression ration of BgTEP1 transcripts in BgTEP siRNA treated snails compared to GFP siRNA treated snails. The relevant mRNA levels were assessed following normalisation with respect to the S19 housekeeping gene in siGFP versus siBgTEP1 injected snails. Means with different letters are significantly different (Kruskall-Wallis test with Dunn post-hoc further adjusted by the Benjamini-Hochberg FDR method, *p* < 0.05, were used). (B) Relative expression of BgTEP1 protein in hemolymph from snails injected with Chol-BgTEP1 siRNA. BgTEP1 protein level was monitored by immunoblotting during 4 days after injection of 10 µg of siRNA in pericardial cavity. Five microliters of hemolymph (15 µg/µL total protein) from each time point (Day 0–Day 4) were run on 7.5% SDS-PAGE and transferred onto Nitrocellulose membrane. BgTEP1 was revealed using a Rat anti-BgTEP1 primary antibody. Amount of BgTEP1 at each day post-siRNA injection was calculated expressed in percentage relatively to BgTEP1 amount in non-injected snails (day 0) using quantitative tools from the Image Lab software v4.0.1. As control, we also monitored the expression of Biomphalysin protein, a protein also expressed in *B. glabrata* hemolymph, using a rabbit anti-Biomphalysin primary antibody.

Once we have demonstrated that cholesterol conjugation promotes siRNA delivery into hemocytes, its efficiency for BgTEP1 gene silencing was tested. BgTEP1 is a thioester-containing protein playing the role in innate immunity as complement-like factor secreted in snail hemolymph (Li et al., 2020a; Mone et al., 2010; Wu et al., 2017). Furthermore, a previous report showed that BgTEP1 could act as an opsonin by binding to the surface of diverse intruders (Portet et al., 2018). Whole snails were microinjected with 10 µg of Chol-TEG siRNA against BgTEP1 or GFP (control). Transcriptional expression analysis of BgTEP1 was focused on circulating hemocytes, normalised with the S19 housekeeping gene and compared with Chol-TEG siGFP injection. Thus, we found a significant reduction in BgTEP1 expression of 4-, 8.5-, and 5.5-fold at day 2, 3 and 4, respectively, when compared to day 1 after siRNA injection (Kruskall-Wallis test: *P* = 0.000054, *df* = 4, *n* = 45, *H* = 24.848) (Fig. 4A). The efficiency of knockdown was also confirmed at the protein level. Indeed, we observed a decrease in plasmatic BgTEP1 protein in hemolymph from nearly 50% at day 1 to 80% at day 2 and 3 and to 90% at 4 days after siRNA injection, while the amount of Biomphalysin used as control for protein loading did not vary significantly (Fig. 4B). Since mRNA level of BgTEP1 was decreased 2 to 4 days after siRNA injection, BgGUA2 snails were exposed to 10 miracidia of *S. mansoni* GH2 at the third day (GFP and BgTEP1). No differences in prevalence and intensity of infection were observed between siBgTEP1- (*P* = 14.7%, *I* = 1, *n* = 34) and siGFP (*P* = 18.6%, *I* = 1.12, *n* = 43) - control injected snails at 16 days following infection (Fisher exact test, *P* = 0.3687).

## Discussion

RNA interference (RNAi) or post-transcriptional gene silencing (*PTGS*) is a biological process highly conserved across animal and plant kingdoms and involved in gene expression or translation inhibition (Almeida & Allshire, 2005; Fire et al., 1998; Plasterk & Ketting, 2000). The RNAi pathway is triggered by short double-stranded RNAs about 21 to 23 base pairs in length which can target complementary mRNA leading to its silencing (Almeida & Allshire, 2005). The RNAi strategy has made possible the investigation of gene function on phenotypic level via the loss of function even if it is transient. The use of dsRNA remains a molecular tool of choice in functional genomics, especially when the CRISPR technology is not yet established for the studied organism. Several modifications can be grafted on short nucleic acid such as 2′ ribose substitutions (Alagia & Eritja, 2016; Allerson et al., 2005; Malek-Adamian et al., 2019) or chemically-modified internucleotide phosphodiester (Alagia & Eritja, 2016; Deleavey & Damha, 2012; Kotikam & Rozners, 2020) to increase gene-silencing efficiency. In addition, achieving high *RNAi* efficiency remains a challenge in particular to target genes in different tissues. Delivery systems for siRNA based on non-viral vectors such as liposomal vesicle or lipid–nucleic conjugation have been developed to remove barriers to the cellular uptake of siRNA linked to its hydrophilic property (De Paula, Bentley & Mahato, 2007; Khalil, Yamada & Harashima, 2018). However, extrapolation of these results or strategies remains difficult in most invertebrates and requires updates on the siRNA administration method (microinjection, soaking, feeding), and the development of delivery systems (viral or non-viral vector) (Selkirk et al., 2012; Whitten, 2019; Zhu & Palli, 2020).

In *Biomphalaria* snail, since the first study reporting the proof of concept for siRNA-mediated gene silencing (Jiang, Loker & Zhang, 2006), only one has been reported comparing the effect of siRNA and long dsRNA soaked juvenile snails on the silencing of the Cathepsin B gene using a cationic polymer vector (Knight et al., 2011). In the present study, we initially focused on the optimization of hemocyte siRNA uptake by conjugating with cholesterol as it was known to be a driving force to enhance cell-penetrating nucleic acid sequences (Zheng & Tai, 2020). Interestingly, the delivery of Chol-siRNAs into HeLa cells has been shown to be very rapid and saturate in less than one hour (Gilleron et al., 2015). After initially reporting that cholesterol conjugation promotes siRNA delivery on hemocytes, we focused on BgTEP1 gene silencing. Indeed, it was shown that this complement factor like protein is largely expressed by snail hemocytes and released in hemolymph (Portet et al., 2018). The several studies of BgTEP1 confer different immune functions to that protein during snail infection. BgTEP1 as described like an antiprotease against different kind of proteinases (Bender & Bayne, 1996), more specifically against a cysteine protease from *S. mansoni* larvae (Fryer, Bender & Bayne, 1996), is also able to bind the surface of different pathogens suggesting a potential opsonin role (Li et al., 2020a; Mone et al., 2010; Portet et al., 2018; Wu et al., 2017). On the basis of these different studies, we speculate that BgTEP gene silencing could increase snail susceptibility to *S. mansoni* parasites.

Nevertheless, no differences were observed in the prevalence and intensity of parasite infection between BgTEP1 siRNA and GFP siRNA treated snails despite the BgTEP1 knock-down at both RNA and protein levels. Several hypotheses can be formulated to explain this unexpected result. The first is functional redundancy between members of the same multigene family. Recently, it was highlighted that BgTEP1 belongs to a multigenic family harboring at least ten other members comprising three C3-like complement factors, one alpha-2-macroglobulin, two macroglobulin complement-related proteins, four insect-like TEP including BgTEP1 and one CD109 molecule (Castillo et al., 2020; Duval et al., 2020). In many invertebrate organisms, several genes encoding TEPs have been identified, fifteen from *Anopheles gambiae*, six from *Drosophila melanogaster* or nine from the tick *Ixodes ricinus* genomes, to name a few examples. If an opsonin role for AgTEP1 could be clearly demonstrated using siRNA (Levashina et al., 2001), the function of *D. melanogaster* TEPs was much more difficult to decipher. Indeed, the importance of TEPs in defense against microbial pathogens has been clearly demonstrated using a quadruple mutant TEP1-4 flies (Dostálová et al., 2017), but single, double or triple mutant flies failed to modify the susceptibility of the insect to different pathogens infection (Bou Aoun et al., 2011). These results suggest that DmTEPs are interchangeable and functionally redundant in pathogen recognition and immune responses. So, we cannot exclude that the function of silenced *B. glabrata* TEP1 could be compensated by another members of the BgTEP family resulting to mask the effect of BgTEP1 knock-down. Moreover, BgTEP1 is not the only thioester-containing protein to interact with the sporocyst tegument, other members such as BgTEP2 and complement C4-like protein display a capacity to bind parasite membrane proteins (Wu et al., 2017).

## Conclusion

We have significantly improved hemocyte siRNA uptake through covalent conjugation of siRNA duplex to cholesterol molecule. For the first time, a snail gene silencing has been successfully monitored in immune cells. This work could be useful to investigate immune gene function in snail hemocytes and to promote new challenges for functional assay development on interfered immune cells.

##  Supplemental Information

10.7717/peerj.10895/supp-1 Figure S1 Intracellular uptake of siRNA in hemocytes After 2 hours Chol-siRNA-cy3 exposure, hemocytes were analyzed by confocal fluorescence microscopy. 500 µL of hemolymph were recovered by head-foot retraction from pool of 3 adult snails and plated for 1 hour on polystyrene chamber slides. Then, hemocytes were incubated 2 hours with 2 µg of Cy3-labeled GFP siRNA conjugated to cholesterol. Alexa-488 phalloidin was used to visualize actin filaments (green) and DAPI for nuclear staining (blue).

10.7717/peerj.10895/supp-2 Data S1 Raw data Four tables with the corresponding raw data for Figures 1C, 2, 3 and 4A.

10.7717/peerj.10895/supp-3 Supplemental Information 1 Uncropped Figure 4B

## Additional Information and Declarations

### Data Availability

The following information was supplied regarding data availability: The raw data concerning results from FACS, RT-QPCR and western blots experiments are available in the Supplemental Files.
