# Supplementary figures and images for: Hemocyte siRNA uptake is increased by 5′ cholesterol-TEG addition in Biomphalaria glabrata, snail vector of schistosome
Source: PeerJ. 2021 Feb 23;9:e10895. doi: 10.7717/peerj.10895 (PMC7908872; doi:10.7717/peerj.10895)

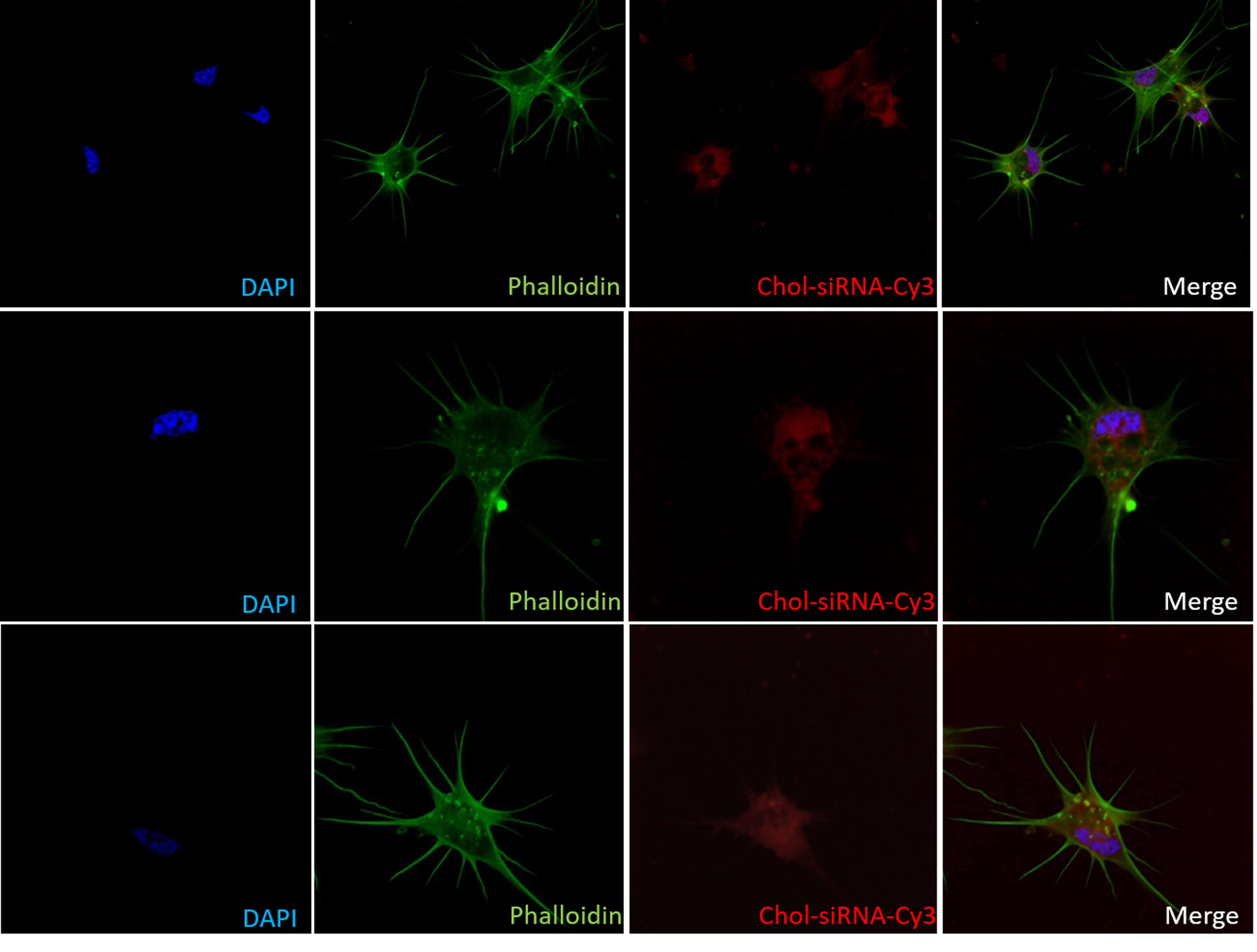

Supplement: Figure S1 — After 2 hours Chol-siRNA-cy3 exposure, hemocytes were analyzed by confocal fluorescence microscopy. 500 µL of hemolymph were recovered by head-foot retraction from pool of 3 adult snails and plated for 1 hour on polystyrene chamber slides. Then, hemocytes were incubated 2 hours with 2 µg of Cy3-labeled GFP siRNA conjugated to cholesterol. Alexa-488 phalloidin was used to visualize actin filaments (green) and DAPI for nuclear staining (blue). [file peerj-09-10895-s001.png]

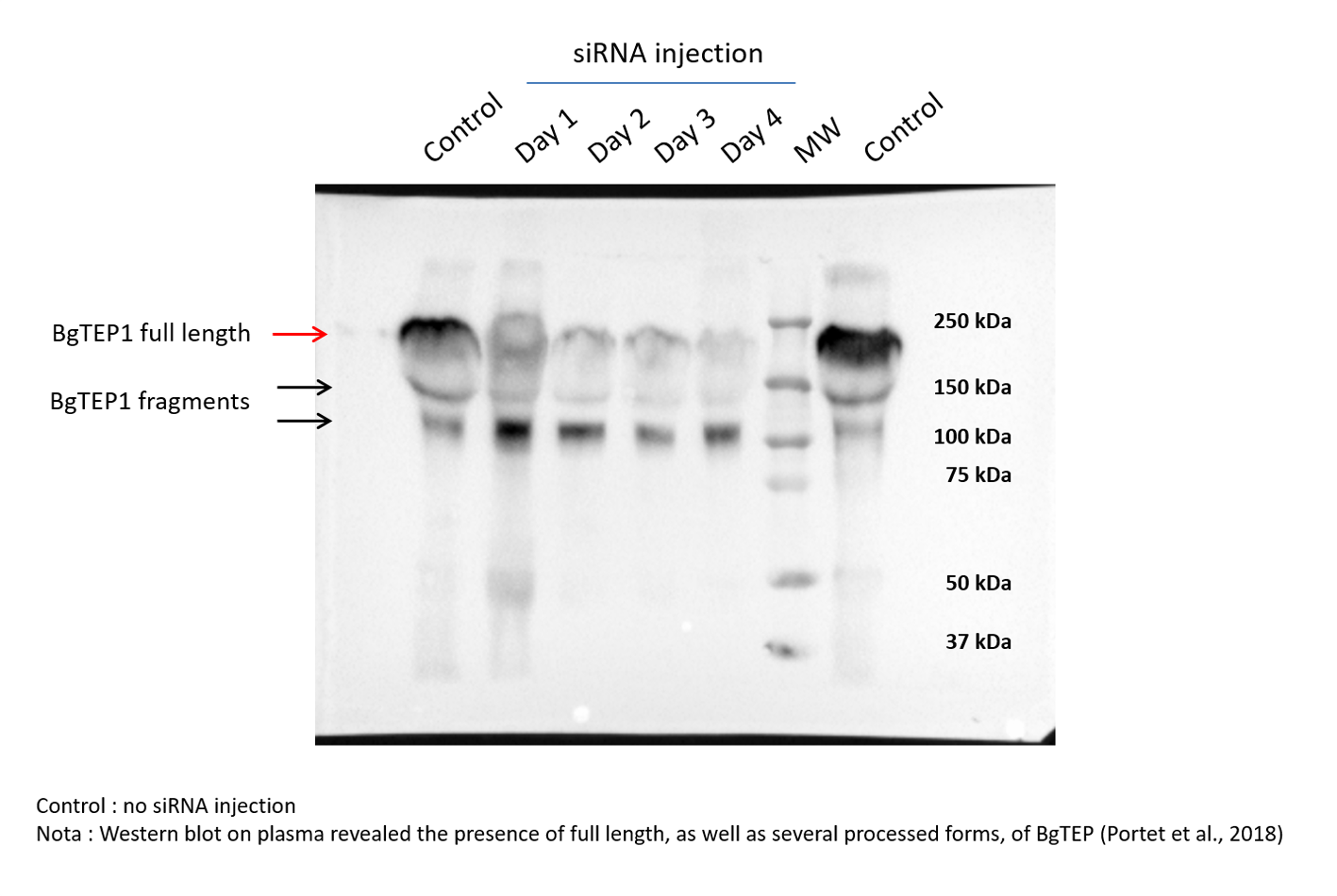

Supplement: Supplemental Information 1 [file peerj-09-10895-s003.png]
